# Asymmetric shape of distal phalanx of human finger improves precision grasping

**DOI:** 10.1038/s41598-021-89791-3

**Published:** 2021-05-17

**Authors:** Ayane Kumagai, Yoshinobu Obata, Yoshiko Yabuki, Yinlai Jiang, Hiroshi Yokoi, Shunta Togo

**Affiliations:** 1 Department of Mechanical and Intelligent System Engineering, Graduate School of Informatics and Engineering, The University of Electro-Communications, Tokyo, Japan; 2 Center for Neuroscience and Biomedical Engineering, The University of Electro-Communications, Tokyo, Japan; 3 Beijing Advanced Innovation Center for Intelligent Robots and Systems, Beijing, China

**Keywords:** Evolution, Anatomy

## Abstract

In morphology field, the functions of an asymmetric-shaped distal phalanx in human finger have only been inferred. In this study, we used an engineering approach to empirically examine the effects of the shape of distal phalanx on the ability of precision grasping. Hence, we developed artificial fingertips consisting of four parts, namely bones, nails, skin, and subcutaneous tissue, that substitute the actual human fingertips. Furthermore, we proposed a method to evaluate the grasping ability of artificial fingers. When a cylindrical object was grasped by an artificial fingertip, a pull-out experiment was conducted. Thus, the asymmetric type was found to be superior in terms of drawing force, holding time, and work of friction than the symmetric type. Our results clearly demonstrate that the asymmetric shape, particularly the mirror-reversed shape of the distal phalanx, improves the ability of precision grasping and suggests that the human distal phalanx is shaped favorably for object grasping.

## Introduction

Researchers in the morphology field inferred from observations and biometrics that there is a close relationship between the morphology of the hand of an animal and its motion capabilities and lifestyle^1^. Hands have played a crucial role in the history of human evolution. Humans exhibit certain morphological features in their hand that are advantageous for grasping and manipulating objects^2,3^. Specifically, the characteristics of human fingertips are regarded as important, and many studies focused on the fingertips.

**Figure 1:**
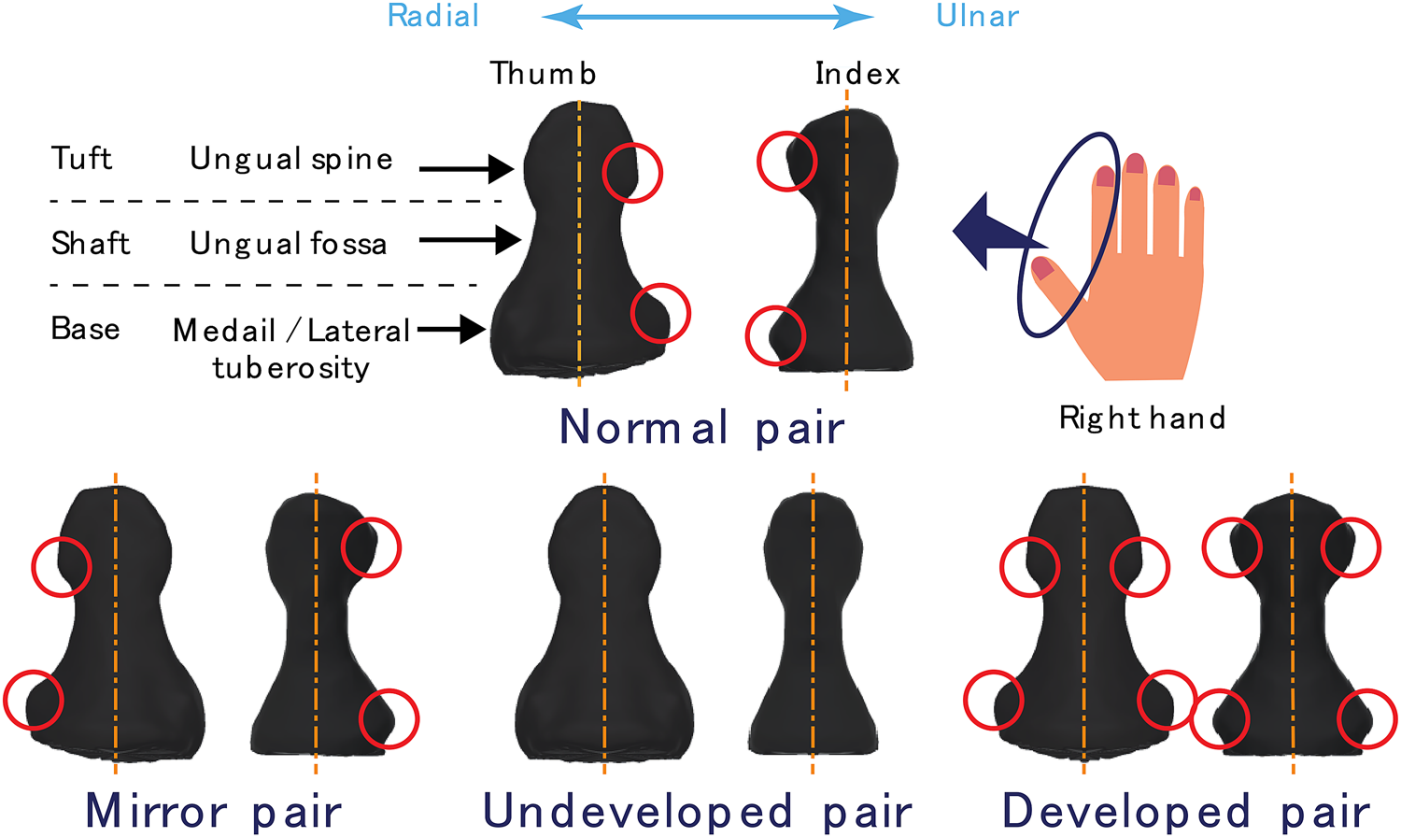
Tested pairs of thumb and index fingertips. The upper row shows a thumb and index distal phalanx of the right hand. Ulnar side of the thumb and radial side of the index finger exhibits ungual spine and medial tuberosity. Additionally, as shown in lower row, we prepared three pairs for comparing the precision grasping ability. The characteristics of the mirror pair are developed on the opposite side of the normal one. Undeveloped pair does not exhibit the developed characteristics on either side. Developed pair exhibits developed characteristics on both sides.

Shimawaki et al. observed that while pressing a plate, the strain on a thumbnail was lower on the ulnar side, which exhibits the characteristics of a developed side, than that on the undeveloped radial side^4^. Similar results were observed during the gripping of a cylindrical object or stroking of a string. The results indicated that this could be due to the asymmetry of skin or the soft tissue inside the finger pad, or distal phalanx^5,6^. The upper row of Fig. 1 shows a distal phalanx of thumb and index finger of a human. Distal phalanx is classified into base, shaft, and tuft (i.e., apical tuft^7,8^ and ungual tuft^9^) from proximal to distal. The distal phalanx of existing great apes (Orangutans, Gorillas and Chimpanzees) exhibits a rod-like shape in general, whereas the human distal phalanx exhibits a developed tuft and distinct boundary^9^. The proximal part of the finger pad is easily deformed and the distal part is not easily deformed due to the developed tuft and the constriction termed as ungual fossa. Tuft has a small protrusion termed as ungual spine^10^. Additionally, with respect to the distal phalanx of the thumb, the protrusion of the tuft on the ulnar side is slightly higher than that on the radial side. According to Pozanski, the prominence of the tuft scale protrusion increases with age^11^. This change in morphology is due to the fact that the ulnar side of the thumb frequently comes in contact with an object and experiences a load^12^. The base also has a protrusion termed as medial / lateral tubercle^9^, and the ulnar side is also more developed. In the case of the index finger, the radial side corresponds to the contact surface because the contact state with the object is reversed when facing the thumb. Therefore, radial protrusion is more prominent than that in the ulnar side. Thus, it is clear that the finger pad and distal phalanx interact while grasping an object, and this causes deformation of the distal phalanx. Therefore, the distal phalanx is potentially involved in the deformation of the finger pad. However, the effects of the morphology of distal phalanx are still unclear. Therefore, we hypothesize that the asymmetry of the distal phalanx leads to a favorable shape for grasping objects.

In the engineering field, several studies focused on soft robot fingers. Shimoga et al*.* reported that robotic hands with soft fingertips can solve certain potential problems^13^. Many designs of soft fingers incorporate nails or endoskeletons to integrate the deformation of the flexible part^14–16^. Controzzi et al*.* focused not only on the skeleton but also on the soft multi-layer structure of the fingers and showed that the grasping by the developed artificial fingers is more stable than that by traditional rigid robotic hands^17^. Yamaba et al. and Shao et al. conducted studies on reproducing the friction characteristics of a finger by using alternative materials^18,19^. Hence, many studies have been conducted on the structure and material properties of the fingertip. However, there is a paucity of studies that consider shape. Or et al*.*^20^ performed a search for an appropriate shape of a soft fingertip to provide stable grasping. However, they did not find any mention of the internal skeletal morphology in any of the extant studies. Thus, understanding the effect of the interaction between the skeleton and soft material on the grasping performance is key to the design of a soft robot finger skeleton.

Based on the viewpoints of morphology and engineering, we examined the effects of asymmetric distal phalanx on object grasping performance to verify the hypotheses on skeletal functions and to gain new insights into the fabrication of robot fingers.

## Results

### Development of the artificial fingertip

**Figure 2:**
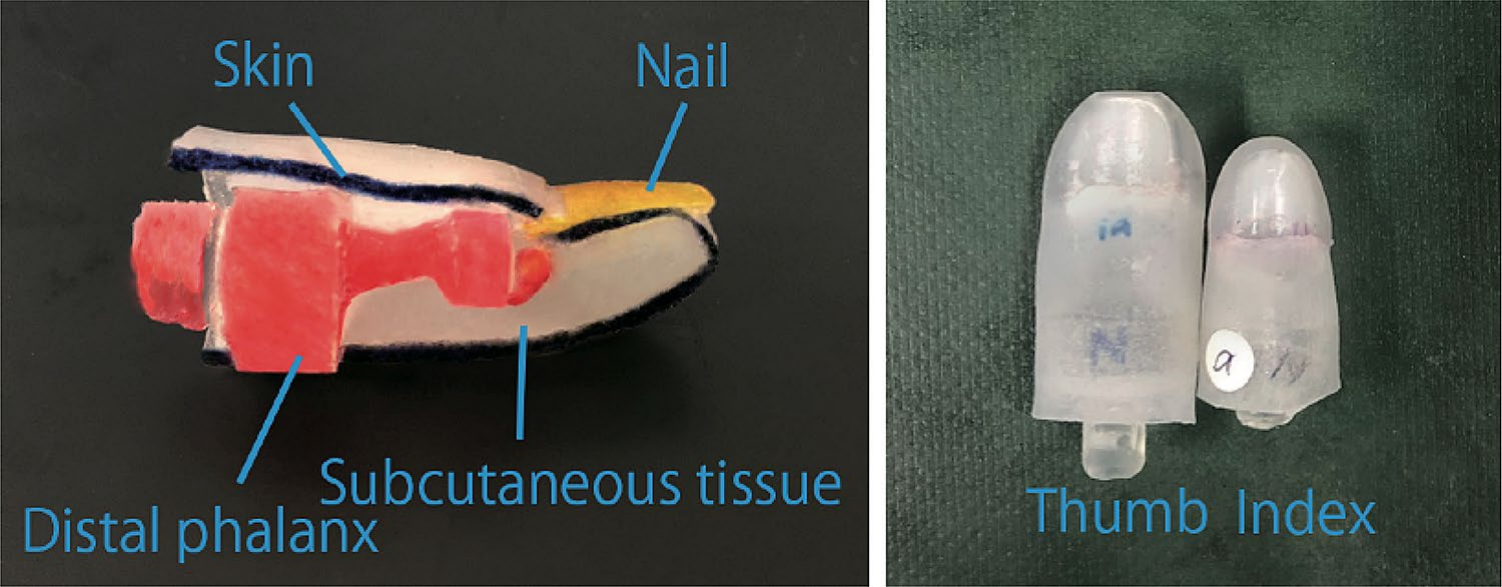
Developed artificial fingertips. Left figure shows the cross section of the developed artificial fingertip. The dummy nail is inserted in the back side of the dummy distal phalanx part. The hole is covered with dummy skin and the space is filled with dummy subcutaneous tissue. The right figure shows artificial thumb and index fingertips viewed from above. The size is approximately the same as that of an adult fingertip.

In this study, we characterized the grasping ability by using artificial fingertips that imitate the structure of human fingers. Our developed artificial fingertip consisted of four elements, namely dummy distal phalanx, nail, skin, and subcutaneous tissue, as shown in Fig. 2. The distal phalanx was 3D-printed from a 3D-scanned human model, which was edited on a 3DCAD system. With respect to a human fingertip, the space between the skeleton and skin is filled with various substances such as blood vessels, fat, and tendons. In this study, all these substances were simplified into a group to understand the effects of the distal phalanx morphology. Therefore, with respect to our artificial fingertips, the definition of the subcutaneous tissue is the internal tissue filling between the skin and distal phalanx, and this differs from the medical definition. All the artificial fingertips were attached to a robot hand (for the right hand).

### Comparison of precision grasping ability

We compared the grasping ability of the thumb and index finger of the right hand, which differ only based on the shape of the distal phalanx. In Fig. 1, the upper row shows the distal phalanxes of the right hand for a normal person viewed from the back. The red circle on the distal phalanx implies that the protrusion is larger than that on the other side. The selected pairs of the distal phalanxes of the thumb and index finger are as follows. The performance was compared among these pairs.

- Normal pair with thumb and index finger exhibiting typical characteristics of the distal phalanx.
- Mirror pair with reversed features of radial and ulnar sides that mirror the normal form along the finger axis.
- Undeveloped pair with undeveloped features on both sides.
- Developed pair with developed features on both sides.

In the experiment, we compared the grasping ability based on precision grasping. Precision grasping is the action of pinching an object between the thumb and index finger and mainly uses the finger pad. Precision grasping accounts for 30% of day-to-day grasping activity^21^.

To evaluate the precision grasping ability, we conducted a pull-out experiment. In this experiment, we measured the maximum drawing force, holding time, and work of friction. The definition of the maximum drawing force is the maximum frictional force required to pull the grasped object from the artificial fingertips. The holding time is the time from when the object is pulled out until the frictional force decreases to 2% of the maximum value. Hence, the work of friction is the workload required to pull out the grasped object from the artificial fingertips. We statistically compared the performances of each pair. Furthermore, we defined that higher values of the aforementioned parameters indicate better grasping ability.

**Figure 3:**
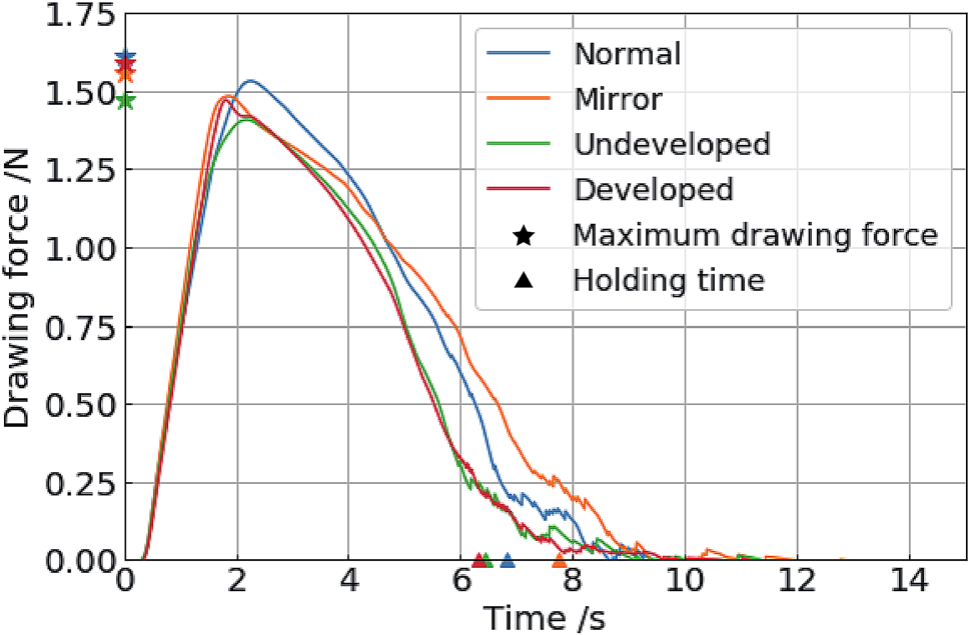
Result of pull-out experiment. Average change with respect to time of the drawing force.

**Figure 4:**
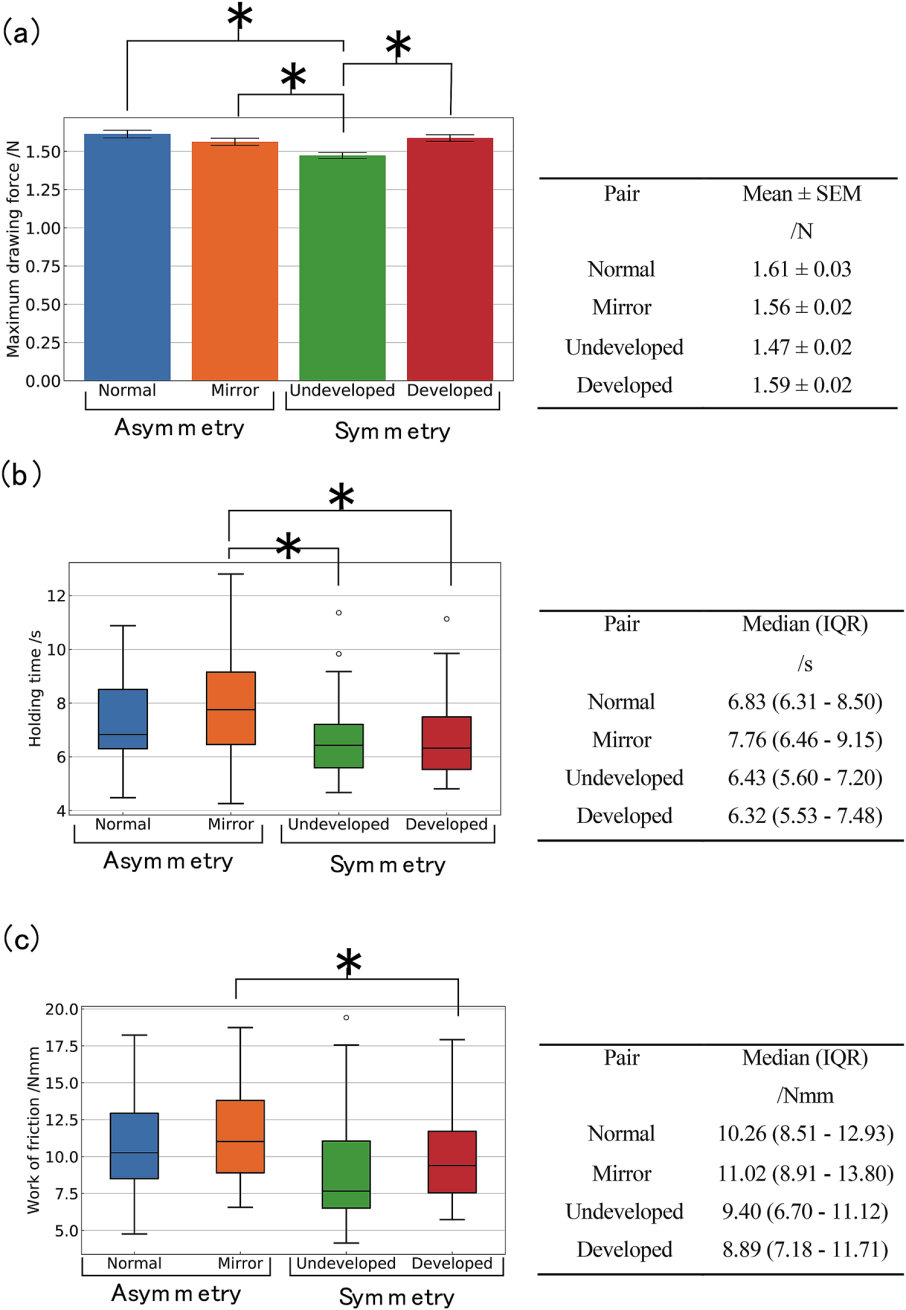
Mean value with a standard error and box plot of each measurement. (**a**) Maximum drawing force, (**b**) holding time, and (**c**) work of friction. Right row shows the mean values and left row shows the median values.

Figure 3 shows the average change over time of the drawing force of each pair. Figure 4 shows the mean with standard error and box plot of each measured quantity. Hence, for the measurement results of these three evaluation criteria, we conducted a statistical analysis. According to the normality test, the samples of undeveloped and developed pairs did not exhibit a normal distribution with respect to holding time and friction work (Table 1, Figure S5). Therefore, the mean value was compared for drawing force and median value was compared for holding time and work of friction. The significance level was *α* = 0.05. Based on the morphological features, the normal and mirror pairs were classified as an asymmetric group, and the undeveloped and developed pairs were classified as a symmetric group. Specifically, mirror pairs exhibited high maximum drawing force, long holding times, and high work of friction. Table 1 shows the *p* value of each statistical test. The asymmetric group was superior to the symmetric group with respect to all the criteria.

**Table 1:**
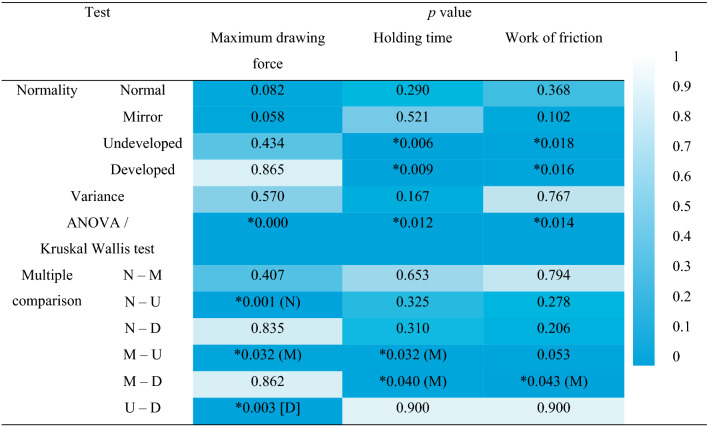
*p* value for each test.

Asterisk (*) indicates *p* < 0.05. In the results with multiple comparisons, () indicates that asymmetry group is superior and [] indicates that there is a difference within the group, and superior pair is in parentheses.

## Discussion

In this study, we hypothesized that the asymmetry of the distal phalanx exhibits a beneficial effect on the grasping ability and measured it by using the three parameters, including maximum drawing force, holding time, and work of friction, as evaluation indexes. Thus, normal and mirror pairs tended to be superior to undeveloped and developed pairs (Fig. 4, Table 1). Specifically, it was observed that the drawing force was higher, the holding time was longer, and the work of friction was higher. Therefore, the results support our hypothesis and suggest that the asymmetry of the distal phalanx in the human finger is effective in improving grasping ability. Furthermore, the results of this experiment suggest that the grasping ability of a soft robot hand can be improved by fabricating asymmetrical bones that are similar to that of humans.

**Figure 5:**
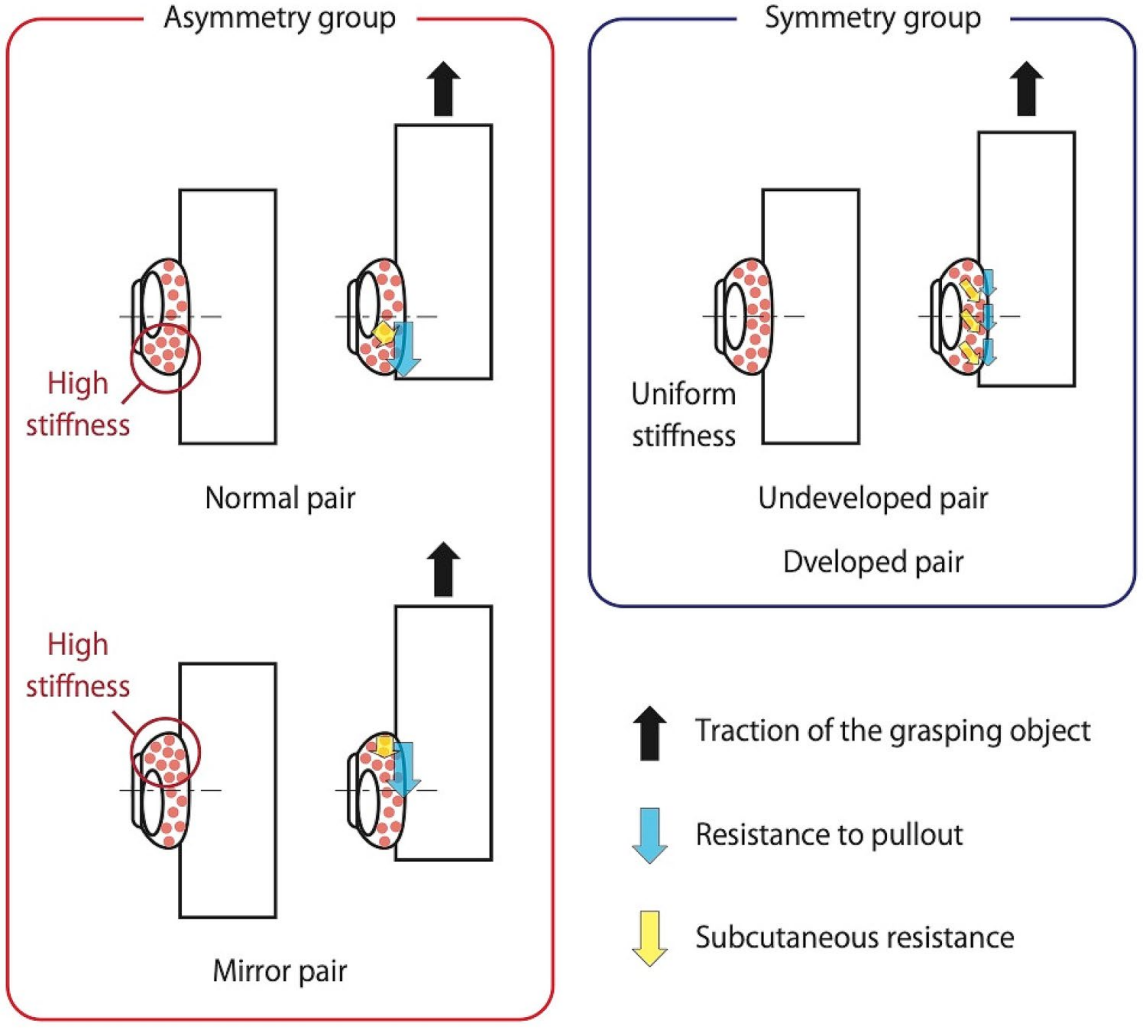
Consideration of the movement of the subcutaneous tissue during the withdrawal of an object. The asymmetric group is expected to be stiffer on one side, while the symmetric group is expected to be more uniformly distributed.

Shimawaki et al. reported that the strain on thumbnail was lower on the ulnar side, exhibiting the characteristics of the development side, than that on the undeveloped radial side^4–6^. Therefore, our experimental results indicated that the grasping ability is improved by biasing the deformation of the finger pad (dummy subcutaneous tissue) on the radial and ulnar sides. Assuming that the grasping object is moving from a stationary state, as shown in Fig. 5, the pressure is evenly distributed in the symmetric group. Furthermore, in the asymmetric group, the mechanical stiffness of the part, where the subcutaneous tissue is concentrated on one side, is increased. Hence, higher stiffness leads to higher reaction force with respect to deformation, and this increases the resistance to the pull-out force (drawing force). This result can be explained by the fact that in Fig. 3, the asymmetric group shows a slower rate of decline in the pull-out force than the symmetric group. Furthermore, the increased resistance during pulling could also increase the holding time. Hence, the results with increased pulling power and longer holding time were reflected via the increase in friction work.

As mentioned in the introduction, the shape of the distal phalanx is considered changeable through development and interaction with the environment^11,12^. Therefore, it is suggested that the shape of the distal phalanx changes from symmetric to asymmetric as the individual develops, which may improve the precision grasping performance. Of course, the shape and degree of asymmetry of the distal phalanxes may vary among individuals. However, if the asymmetric shape changes the distribution of stiffness and improves the grasping force, as suggested in the present study, then it would benefit the grasping as long as the distal phalanx is asymmetric. The relationship between differences in the degree of asymmetry of the distal phalanx and differences in grasping ability that depend on individual characteristics should be investigated in the future.

As the purpose of this study was to investigate the characteristics of human distal phalanxes, the shapes of the models were changed without deviating from their size. However, considering the application to soft robot hands, an optimal shape to improve the precision grasping ability may exist, for example, an exaggerated asymmetry structure. We have considered only the task of withstanding the pulling force in one direction. However, human fingertips perform this task under several conditions in daily life. Therefore, it is possible that exaggerating the asymmetry reduces the performance of other tasks. In this study, there was no statistically significant difference between the normal pair and mirror pair. However, the difference between the asymmetric group and mirror pair was likely to occur. The shape characteristic of the mirror pair is reversed when compared to that of the normal pair. Thus, the normal pair is expected to be advantageous for the pull-out in the direction of gravity. We performed extraction only in the opposite direction of gravity without changing the relative position of the artificial finger and cylindrical object. In the future, it is necessary to consider the effects of the distal phalanx morphology under various conditions such as the pulling direction and the position and shape of the object. Thus, it is important to investigate the relationship between distal phalanxes that deviate from the typical shape and performance on various tasks.

An important challenge also remained from the material aspect. In this study, we developed an artificial fingertip that qualitatively replicates the characteristics of a human finger, i.e., the skin is harder than the inner tissues of the finger, and investigated the effect of the shape of distal phalanxes on the precision grasping performance. All the materials used for each artificial fingertip were the same. Therefore, the results of this study are dependent on the parameters of the shape of the distal phalanxes. However, the results may change if different materials are used. There is possibility that the viscoelastic properties of real and artificial fingertips are different. Quantitative comparison of the viscoelastic properties of real and artificial fingertips is an important issue for the future.

The analysis of the deformation of the finger pad during the movement of the grasping object is also a future task. By observing the deformation of the finger pad, while grasping the object and considering the interaction between the skeleton and the soft cover, it will be possible to develop a better performing finger.

The remaining question is how the asymmetry of the human distal phalanx has been shaped. Two possibilities are innate and/or use-dependent mechanisms. In the innate mechanism, the shape of the distal phalanx is genetically encoded. In contrast, in use-dependent mechanisms, the distal phalanx gradually grows into an asymmetrical shape with daily use. In this study, an artificial fingertip was made from a fully developed distal phalanx, demonstrating the advantages of an asymmetric shape in precision grasping. Therefore, it is difficult to infer from the results of this study how the asymmetry of the distal phalanx is shaped. The results lead to the hypothesis that the asymmetrical deformation of the subcutaneous tissue under pressure is the mechanism that improves the grasping ability. If this asymmetrical deformation of the subcutaneous tissue is advantageous in the process of evolution, it is possible that the shape of the distal phalanx is genetically encoded such that the subcutaneous tissue deforms asymmetrically. By contrast, a previous study reported that the shape of the distal phalanx changes with development and usage^11^. Therefore, both innate and use-dependent mechanisms may influence the morphology of the distal phalanx, which may lead to improved object grasping ability. To answer this question, it is necessary to investigate the development process, the change process in distal phalanx shape, and the change process in precision grasping ability systematically.

## Methods

### Procedure for manufacturing an artificial fingertip

We developed an artificial fingertip based on the anatomy by integrating four parts, namely distal phalanx, nail, skin, and subcutaneous tissue. A method for manufacturing each part that constitutes the artificial fingertip is described below. A simplified diagram of the fabrication process is presented in Fig. S2 in the supplementary methods section. The dimensions were determined based on the ratios of finger parts and dimensional data of Japanese fingers surveyed by Yoshida et al. (Table S1)^22,23^. Furthermore, the dimensions and masses of the fabricated fingers were measured (Fig. S3).

#### Dummy distal phalanx

A clear resin was used as the material dummy distal phalanx. First, we scanned the distal phalanx of a normal thumb and index finger and developed a skeletal model by using a 3D scanning software (ReCap Photo, Autodesk Inc., US). The scanned 3D data was modified with a CAD software (Fusion360, Autodesk Inc., US) to create four types of distal phalanxes. A groove for inserting the nail was provided on the back side of the distal phalanx, and a base was provided on the bottom. A shaft for attaching an artificial finger to a dedicated hand was provided on the proximal surface of the base. Finally, we printed the completed 3D model using an optical 3D printer (Form2, Formlabs Inc., US).

#### Dummy nail

We chose commercially available acrylic nail tips as dummy nails. The nail tip was cut as per the total length of the exposed area and insertion area.

#### Dummy skin

The material of dummy skin corresponded to A60 silicone (TSE-3466, Tanac Co., Ltd., Japan). The liquid main agent and curing agent were poured into a beaker at a weight ratio of 10:1 and stirred. The mixed A60 silicone was painted to a finger-shaped mold and heated on a hot plate until it solidified. The process was repeated three times to form a film, which was used as a dummy skin. Finally, the artificial skin was peeled off from the finger mold, a cut was made, and the nail was fastened.

#### Dummy subcutaneous tissue

The material for the dummy subcutaneous tissue was E30 silicone (TSG-E30, Tanac Co., Ltd., Japan). E30 silicone is softer than A60 silicone. Furthermore, in real human skin, the outer layer of the skin is harder^24^. The liquid main agent and curing agent were poured into a beaker at a weight ratio of 1:1 and stirred. The mixed silicone was poured into the dummy skin with the dummy nails, and the dummy distal phalanx was pushed into it. This was left in the incubator at a constant temperature (36.5° C in this study) for several tens of minutes until the reaction converged.

### Apparatus

The items and equipment necessary for the experiment are as follows.

- Four types of pairs of artificial thumb and index finger, each with a different shape of the distal phalanx (normal, mirror, undeveloped, developed). Two thumbs and two index fingers are prepared for each type.
- An experimental robot hand (right hand) for attaching artificial fingers. It is designed based on a 2-DOF simple prosthetic hand produced by Jiang et al.^25^. The MP joint has 1-DOF and performs a gripping operation with a servo motor (HP-DH16-FTD, HYPERION HK Ltd., Hong Kong). This hand was position-controlled using a commercially available servo-controller. Before the experiments, we measured the pinch force of the robotic hand. When the knob on the servo controller was turned to the maximum value, the robot hand generated a 3-N pinch force. The finger part is cut off at the tip and has a shaft and boss for attaching an artificial finger. The opening angle formed between the thumb and the index finger when in contact without grasping anything (when the thumb and index finger overlapped in the top view) in both the lateral and front views was approximately 45°. The angles in the lateral view were based on a hand that mimics the human hand designed by Jiang et al*.*^25^. The angles in the front view were carefully aligned based on the actual 3D data of the precise grasping posture (Figure S4).
- A commercially available servo controller and battery for driving servo.
- Fixing tools for the robot hand.
- An acrylic cylindrical object, which is the grasping target gripped by the fingers. The diameter of the cylinder is 21 mm.
- Baby powder to remove the viscosity of the artificial finger surfaces
- Tensile tester (MC-2150, A&D Inc., Japan) and analysis software (MSATLite, A&D Inc., Japan) for the pull-out experiment. The tester is controlled in position and can measure the displacement of the object that is pulled out. Furthermore, the tester can measure the load on the towing object at regular intervals.

### Procedure

**Figure 6:**
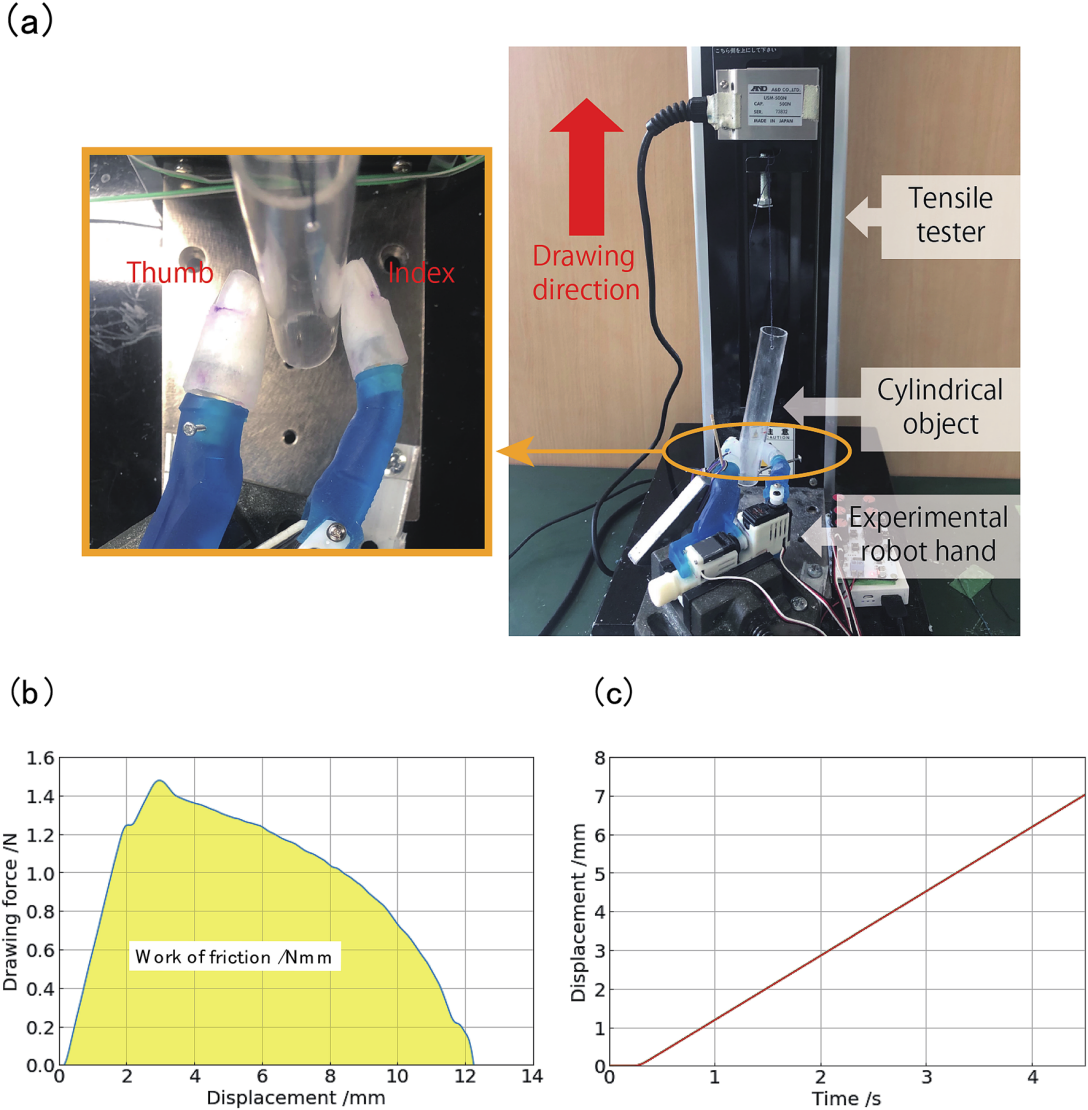
Pull-out experiment. (**a**) Experimental setup. The right figure shows the state of precision grasping of the artificial fingertips during an experiment viewed from above. As shown in left figure, a cylindrical object is lifted up by the tensile tester. (**b**) Obtained waveforms. Representative examples of waveforms obtained during the pull-out experiment. (**c**) Time-displacement plot resulting from the pull-out experiment. The time variation of the displacement is constant.

Before starting the experiment, baby powder, which contains talc as its main ingredient was applied to the artificial finger surface. As Shao et al*.*^19^ reported, the viscosity of the artificial finger surface, i.e., silicone rubber, has a quite large effect on the frictional properties, which is a factor that makes it difficult to show the difference in performance due to the shape of the distal phalanx inside the finger. Therefore, as in Shao et al*.*^19^, we reduced the effect of viscosity on grasping performance by applying powder containing talc to each finger model in approximately the same amount. The powder was applied using a puff, with a volume of approximately 0.003–0.004 g for the thumb and 0.002–0.003 g for the index finger. As described in the Method section, the skin of each artificial finger was made of the same material and manufacturing process, thus the difference in contact conditions between the fingers was considered to be small. As shown in Fig. 6a, a cylindrical object was suspended with a thread and is held by the hand attached with artificial fingers. During the pull-out experiments, the experimenter kept the knob on the servo-controller at the maximum value. The cylinder was pulled up using a tensile tester. The vise holding the robot hand was attached to the base of the tensile tester by screws, and the hand mounting conditions were controlled between trials. The cylinder was marked at the height of contact with the artificial fingers. The experimenter visually confirmed that the contact between the cylinder and the artificial fingers was the same at each measurement, and the thread was not stretched across trials. The pull-out direction was from the ulnar to the radial side of the hand. The time, displacement (lifting distance), and frictional force required for the cylindrical object to slip through the finger were measured from the start of the lifting process. We fabricated two thumb and index fingers to make four combinations for each pair. Subsequently, the measurement was performed 10 times and a total of 40 data points of the pull-out experiment were obtained. Figure 6b shows the correspondence between the amount of lift of the cylindrical object and drawing force. This illustrates the representative data of the experiment. Given that the work of friction was not directly measured, it was calculated by numerically integrating the frictional force with respect to the displacement by using the Simpson's method. The displacement of the cylinder is constant during the pull-out experiment (Fig. 6c). Moreover, we confirmed that the variability of the initial angles between the grasped cylinder and the central axis of the index finger was small in an additional experiment (see the Supplementary results). Therefore, we could accurately repeat the measurement experiments.

### Data analysis

Multiple comparisons were performed for the three measured quantities. In this experiment, it was necessary to compare all the groups. The data sample size was *n* = 40.

First, we tested the normality of the sample population via the Shapiro–Wilk test. The significance level of all the tests we used corresponded to *α* = 0.05. With respect to the maximum drawing force, all groups followed normal distribution. Conversely, undeveloped and developed pairs did not exhibit normal distributions with respect to holding time and work of friction. Subsequently, the homoscedasticity between all groups was tested via Bartlett's test or Levene’s test, and the variance was equal for all the criteria. Therefore, the maximum drawing force should be tested by a parametric test, and the holding time and work of friction should be tested by a non-parametric test. Before testing multiple comparisons, we confirmed that the null hypotheses of "no difference between groups" of ANOVA for maximum drawing force and that of the Kruskal–Wallis test for holding time and work of friction were rejected. Next, we used Tukey’s test for the former, and Steel–Dwass test for the latter. Given that it is not possible to predict in advance as to which group is better, both tests were two-tailed tests.

## Supplementary Information

Supplementary Information.
