# Supplementary material for: Asymmetric shape of distal phalanx of human finger improves precision grasping
Source: Sci Rep. 2021 May 17;11:10402. doi: 10.1038/s41598-021-89791-3 (PMC8128879; doi:10.1038/s41598-021-89791-3)
Supplement: Supplementary file 1 — Supplementary Information. [file 41598_2021_89791_MOESM1_ESM.docx]

Asymmetric shape of distal phalanx of human finger improves precision grasping

Ayane Kumagai^1*^, Yoshinobu Obata^1^, Yoshiko Yabuki^1^, Yinlai Jiang^2,3^, Hiroshi Yokoi^1,2,3^, and Shunta Togo^1,2^

^1^Department of Mechanical and Intelligent System Engineering, Graduate School of Informatics and Engineering, The University of Electro-Communications, Tokyo, Japan.

^2^Center for Neuroscience and Biomedical Engineering, The University of Electro-Communications, Tokyo, Japan.

^3^Beijing Advanced Innovation Center for Intelligent Robots and Systems, Beijing, China.

*Corresponding author

E-mail: [a.kumagai@hi.mce.uec.ac.jp](mailto:a.kumagai@hi.mce.uec.ac.jp) (AK)

**Supplementary information**

### **Supplementary methods**


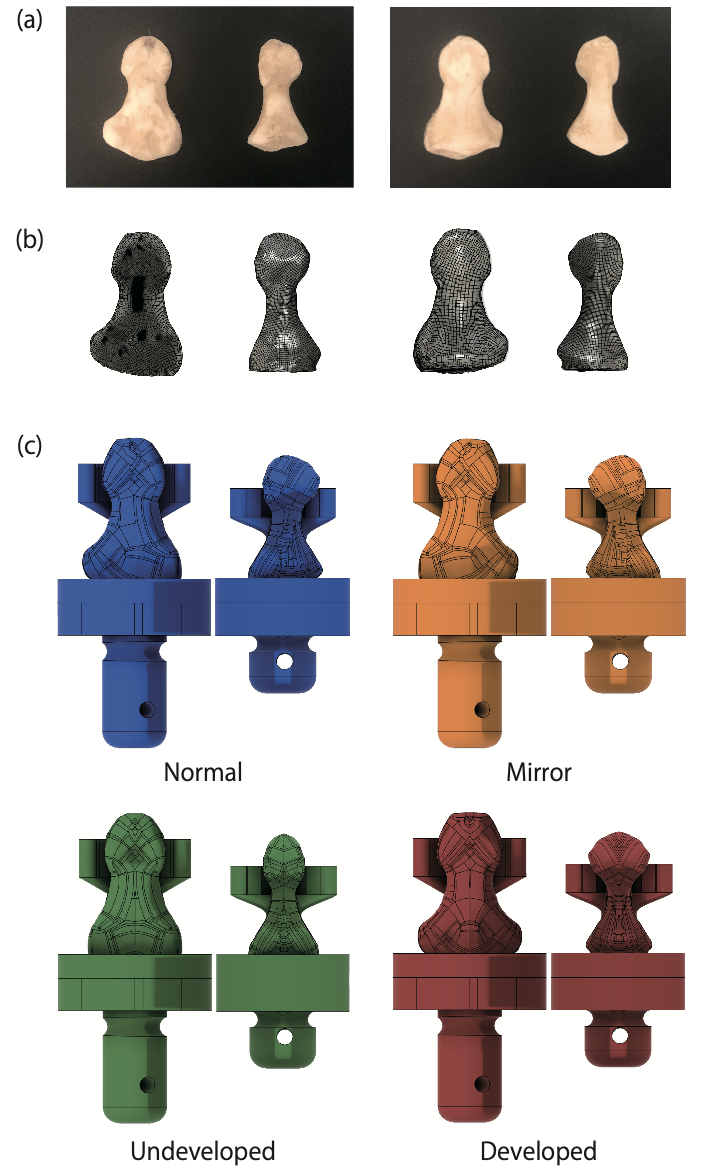


**Figure S1 | From the 3D scan of a human body model to the design of the distal phalanx parts.** (a) Distal phalanxes of the human model, (b) their 3D data, and (c) distal phalanx parts designed based on the 3D data.
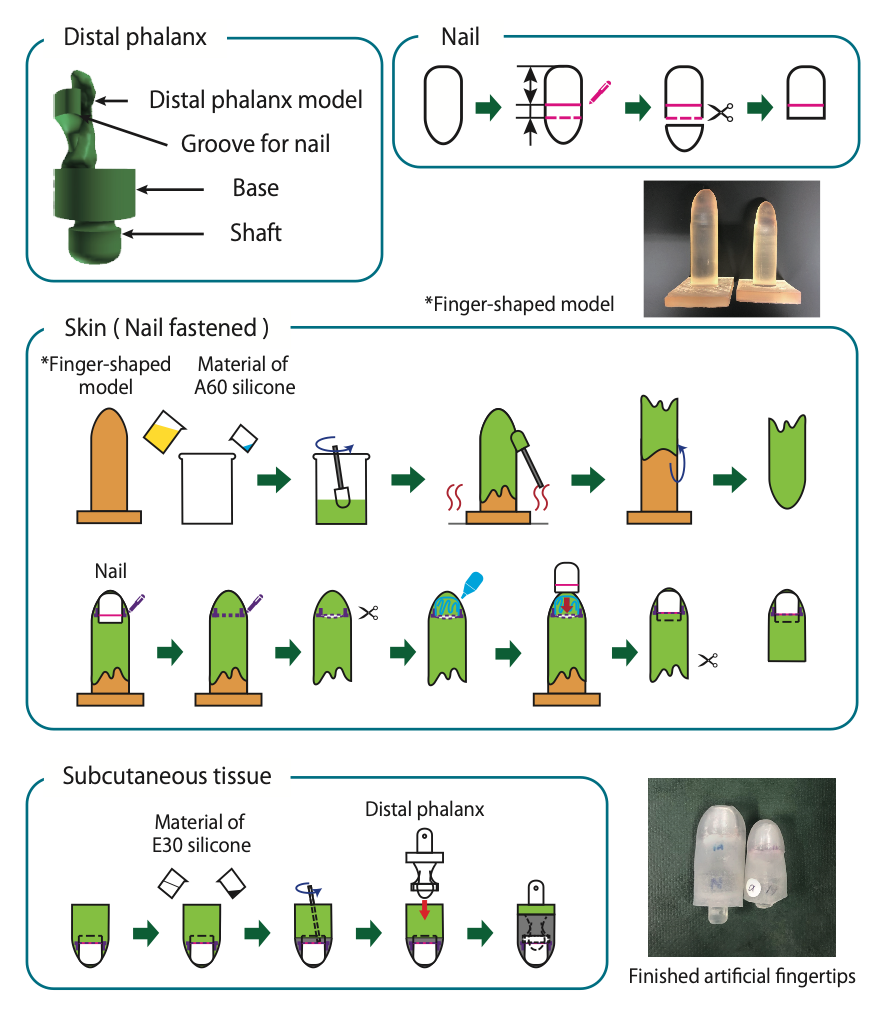


**Figure S2 | Artificial finger manufacturing process.**

**
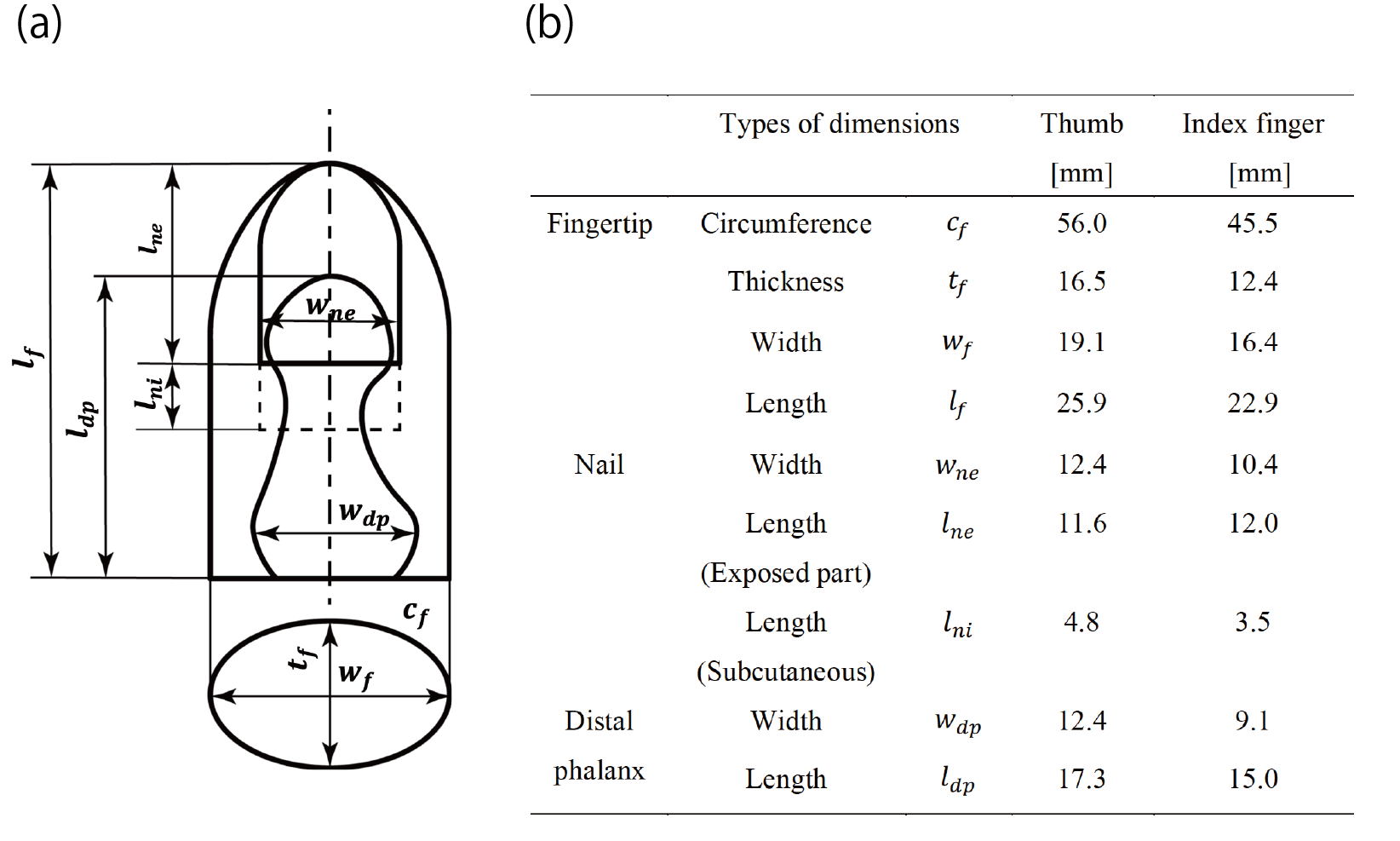
**

**Figure S3 | Setting the dimensions of the artificial fingertip (target dimensions).**

**Table S1 | Dimensions and mass of manufactured artificial finger (outer shape)**

|  | Type | Width $w_{f}$  [mm] | Length $l_{f}$  [mm] | Thickness $t_{f}$  [mm] | Mass  [g] |
| --- | --- | --- | --- | --- | --- |
| Thumb | Normal | 20.7 | 28.3 | 16.6 | 4.93 |
|  |  | 20.7 | 28.3 | 16.3 | 4.94 |
|  | Mirror | 20.7 | 28.3 | 16.5 | 4.94 |
|  |  | 21.0 | 28.3 | 16.5 | 4.91 |
|  | Undeveloped | 20.6 | 28.6 | 16.0 | 4.95 |
|  |  | 20.8 | 28.4 | 16.3 | 4.96 |
|  | Developed | 19.4 | 28.2 | 16.7 | 4.95 |
|  |  | 19.5 | 28.3 | 16.6 | 4.95 |
|  | Mean (STD) | 20.4 $\pm$ 0.6 | 28.3 $\pm$ 0.1 | 16.4 $\pm$ 0.2 | 4.94 $\pm$ 0.02 |
| Index finger | Normal | 16.4 | 24.5 | 14.8 | 2.78 |
|  |  | 16.4 | 24.6 | 14.1 | 2.77 |
|  | Mirror | 16.4 | 24.6 | 14.0 | 2.79 |
|  |  | 16.4 | 23.8 | 13.0 | 2.73 |
|  | Undeveloped | 16.2 | 23.8 | 13.6 | 2.74 |
|  |  | 16.7 | 24.8 | 13.6 | 2.76 |
|  | Developed | 16.8 | 24.3 | 14.2 | 2.74 |
|  |  | 17.0 | 24.8 | 13.4 | 2.73 |
|  | Mean (STD) | 16.5 $\pm$ 0.3 | 24.4 $\pm$ 0.4 | 13.8 $\pm$ 0.6 | 27.6 $\pm$ 0.02 |

In this paper, the main dimensions of the artificial fingertip are defined as follows (Fig. S3 (a)).

・Circumference of fingertip $c_{f}$: The circumference of the *base part of the fingertip.

・Thickness of fingertip $t_{f}$: The short diameter of the *base part of the fingertip.

・Width of fingertip $w_{f}$ : The maximum width of the *base part of the fingertip (the cross-section of the fingertip is an ellipse and its long diameter).

・Length of fingertip $l_{f}$ : The length at the midline from the distal end of the *base part to the distal end of the fingertip.

・Width of nail $w_{ne}$ : The maximum width of the exposed part of the nail.

・Length of nail $l_{ne}$ (exposed part): Length at the center line of the exposed part of the nail.

・Length of nail $l_{ni}$ (subcutaneous) : Length at the center line of the subcutaneous part of the nail.

・Width of distal phalanx $w_{dp}$ : The maximum width of **distal phalanx base.

・Length of distal phalanx $l_{dp}$ : Length of the proximal to distal end of the distal phalanx.

(*base part of fingertip implies “Base” of fig. S1, **distal phalanx base implies “Base” of Fig. 1)

The target dimensions calculated from the data of the previous study are shown in Fig. S2 (b) [22] [23]. The dimensions and total masses of the parts of the finished fingertips, which are subject to errors in production, are measured and shown in Table S1.

**Table S2 | Asymmetry rate for each pair (asymmetry rate = ulnar volume/radius volume)**

|  | Thumb | Index |
| --- | --- | --- |
| Normal | 1.021  (277.85$\mathrm{mm}^{3}$: 283.71$\mathrm{mm}^{3}$) | 0.788  (181.22$\mathrm{mm}^{3}$: 142.76$\mathrm{mm}^{3}$) |
| Mirror | 0.979  (283.71$\mathrm{mm}^{3}$: 277.85$\mathrm{mm}^{3}$) | 1.269  (142.76$\mathrm{mm}^{3}$: 181.22$\mathrm{mm}^{3}$) |
| Undeveloped | 1  (277.85$\mathrm{mm}^{3}$: 277.85$\mathrm{mm}^{3}$) | 1  (142.76$\mathrm{mm}^{3}$: 142.76$\mathrm{mm}^{3}$) |
| Developed | 1  (283.71$\mathrm{mm}^{3}$: 283.71$\mathrm{mm}^{3}$) | 1  (181.22$\mathrm{mm}^{3}$: 181.22$\mathrm{mm}^{3}$) |


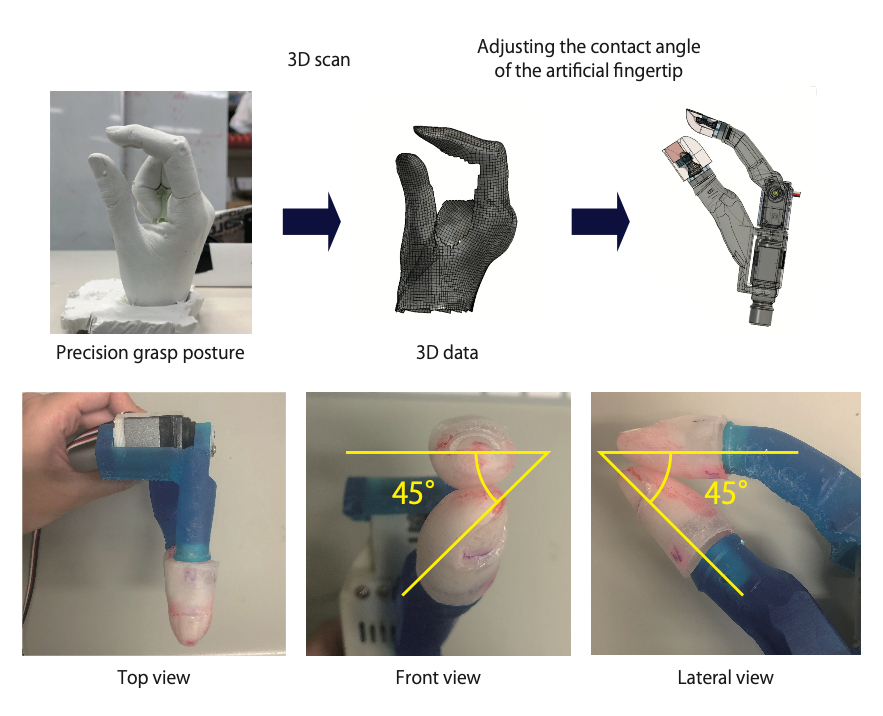


**Figure S4 | Precision grasping posture of an experimental robot hand.** First, a plaster of the precision grasping posture was scanned and then the plaster was converted into 3D data via 3D scanning. The angle of the artificial fingertip attachment of the robotic hand was adjusted as per the fingertip contact state of the 3D data. The opening angle formed between the thumb and the index finger when in contact without grasping anything (when the thumb and index finger overlapped in the top view) in both the lateral and front views was approximately 45°.

### **Supplementary results**


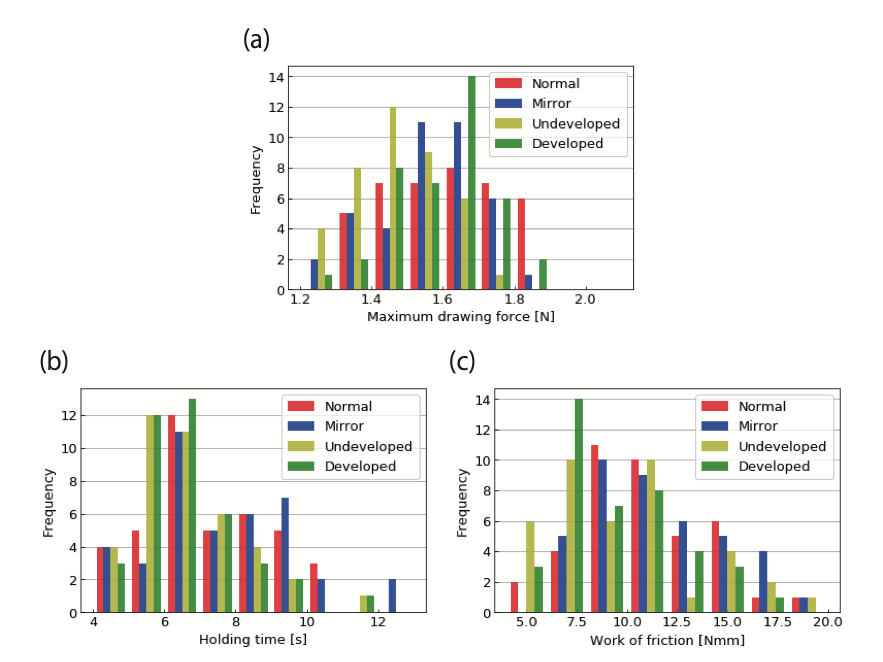


**Figure S5 | Histograms for each criterion.**  Histogram for each measurement – (a) maximum drawing force, (b) holding time, and (c) work of friction. Each frequency is indicated by the following color – red: normal, blue: mirror, yellow: undeveloped, and green: developed pair.

Asymmetry group does not exhibit normal distribution with respect to holding time and work of friction. Asymmetric groups do not perform consistently when compared to normally distributed symmetric groups. Figures 3 (b) and (c) show that the frequencies are concentrated on values that are lower than the mean value in the asymmetry pair. In symmetry pairs, higher values can be realized when compared to those in asymmetric groups. However, this is rare. With respect to maximum drawing force, all pairs exhibited normal distribution.

**Table S3 | Result of the supplementary experiment. Angle between the cylinder and the central axis of the index finger.**

|  | Normal | Mirror | Undeveloped | Developed |
| --- | --- | --- | --- | --- |
| Mean ± STD /° | 90.5 $\pm$ 0.76 | 90.75 $\pm$ 0.91 | 89.55 $\pm$ 0.76 | 90.75 $\pm$ 0.91 |

As an additional experiment, the angle between the cylinder and the central axis of the index finger was measured with a fixed-point camera. The initial angles of each pair were measured 20 times each, with the traction direction set at 90$^{\circ}$. The results were as follows. Although there was some bias, the initial angles of all pairs were within the range 90 ± 1$^{\circ}$. The standard deviations of all pairs were also small, within 1$^{\circ}$, and the reproducibility between trials was high. In addition, in the pull-out experiment described in the manuscript, the initial positioning of the traction part of the tensile tester was mechanically restored by the system after each trial, so there was no difference in the initial positioning between trials or between pairs. Based on the above results, the starting kinematics were repeated with high accuracy in successive trials. Moreover, there was small systematic difference in the initial positioning between different phalanx models.
